# A longitudinal study on the correlation between symptom clusters and disease-specific health status in older adults with chronic obstructive pulmonary disease during pulmonary rehabilitation

**DOI:** 10.3389/fpubh.2026.1720782

**Published:** 2026-01-20

**Authors:** Jinghua Yang, Na Xu, Mengyao Liang

**Affiliations:** 1 Department of Nursing, Affiliated Jiangyin Hospital of Nantong University, Jiangyin, China; 2 Department of Respiratory Critical Medicine, Affiliated Jiangyin Hospital of Nantong University, Jiangyin, China; 3 Department of Nursing, The Sixth People’s Hospital of Nantong, Nantong, China

**Keywords:** aged, health status, longitudinal studies, pulmonary disease, chronic obstructive, symptom clusters

## Abstract

**Objective:**

The present study aims to use a longitudinal design to clarify the dynamic evolution trajectory of symptom clusters in older adults with chronic obstructive pulmonary disease (COPD) during pulmonary rehabilitation and to analyze the longitudinal association between the evolution of these symptom clusters and patients’ disease-specific health status.

**Methods:**

A prospective longitudinal study design was employed. From January 2025 to September 2025, 184 older COPD patients receiving pulmonary rehabilitation were enrolled. Data were collected at baseline (T0), 4 weeks (T1), and 12 weeks (T2) of rehabilitation using a general information questionnaire, the Memorial Symptom Assessment Scale (MSAS), and a COPD rehabilitation-specific symptom supplementary assessment tool. Symptom clusters were extracted using exploratory factor analysis. Spearman correlation analysis was used to examine the association between symptom cluster severity and CAT (COPD Assessment Test) disease-specific health status scores.

**Results:**

The symptom cluster structure underwent significant evolution throughout the rehabilitation process. At T0, five core symptom clusters were extracted, dominated by a respiratory-metabolic cluster (shortness of breath, cough, sputum, fatigue), which accounted for a cumulative variance contribution rate of 61.32%. At T1, the symptom clusters reconfigured, with the emergence of a new cluster characterized by post-exertional malaise (muscle soreness, edema). By T2, the structure consolidated into four symptom clusters, with chronic fatigue and social function inhibition (social anxiety, loss of confidence) becoming the core manifestations. Correlation analysis revealed a dynamic change in the impact of symptom clusters on health status: at T0, the psychological-emotional symptom cluster showed the strongest correlation with CAT scores (r = 0.714, *p* < 0.001); by T2, the social function inhibition cluster became the primary influencing factor (r = 0.691, p < 0.001), indicating a shift in the dominant association from psychological to psychosocial factors over time.

**Conclusion:**

The findings of this study demonstrate that the evolution of symptom clusters in older COPD patients during pulmonary rehabilitation follows a distinct temporal pattern: progressing from a predominance of respiratory-metabolic symptoms, to the prominence of post-exertional malaise, and finally solidifying with psychosocial symptoms as the core manifestation. The primary impact of symptom clusters on health status also shifts across rehabilitation stages. From a public health perspective, our results provide a rationale for integrating symptom cluster assessments into community-based geriatric care and long-term support systems. This approach is vital for promoting healthy aging, as it enables proactive interventions tailored to the evolving needs of older adults with COPD, thereby optimizing resource allocation and improving the sustainability of care for this large patient population.

## 1 Introduction

According to data from the World Health Organization, Chronic Obstructive Pulmonary Disease (COPD) has become the third leading cause of death globally, imposing a significant disease burden on healthcare systems worldwide (1). As of recent years, the number of COPD patients in China has reached nearly 100 million, accounting for approximately one-quarter of the total global COPD patient population. The prevalence among the older adults is significantly higher than in other age groups and shows a clear increasing trend with age (2). With advancing age, the physiological decline in lung function in older adults, combined with the pathological damage from COPD, leads to a sharp reduction in respiratory functional reserve. Furthermore, older adults patients often have multiple comorbidities (such as cardiovascular disease, osteoporosis, diabetes, etc.), which further exacerbate the complexity and management difficulty of the disease. The clinical manifestations of COPD are heterogeneous, with common symptoms including persistent cough, sputum production, shortness of breath or dyspnea. These symptoms severely impact patients’ daily activity capacity and social participation, leading to a significant decline in disease-specific health status (3, 4).

Pulmonary Rehabilitation (PR), jointly recommended by the American Thoracic Society and the European Respiratory Society, is a core non-pharmacological treatment strategy and is rated as Grade A evidence in the management of COPD by multiple international guidelines (5). Pulmonary rehabilitation is defined as “a comprehensive intervention program based on a thorough patient assessment, encompassing exercise training, health education, and behavior change, aimed at improving the physical and psychological condition of patients with chronic respiratory disease and promoting the long-term adherence to health-enhancing behaviors” (6). Exercise training, as a key component of pulmonary rehabilitation, has demonstrated clear benefits in improving exercise tolerance in COPD patients. Long-term, regular exercise training can enhance aerobic and anaerobic metabolism in muscle cells, ameliorate dyspnea, and increase exercise endurance (7). Studies have shown that after 6 weeks of structured pulmonary rehabilitation training, COPD patients exhibit significant improvements in exercise capacity indicators such as load endurance time, 6-min walk test distance, and peak oxygen consumption (8, 9).

The concept of Symptom Cluster was first proposed by Dodd et al., referring to a set of multiple interrelated and co-occurring symptoms. These symptoms have intrinsic connections and collectively influence patient outcomes as a whole (10). In the field of COPD, symptom clusters are typically composed of both physiological and psychological symptoms, characterized by their dynamic and temporal nature. Research indicates (11–13) that COPD patients experience not only core respiratory symptoms (such as shortness of breath, cough, and sputum) but also commonly suffer from systemic symptoms like fatigue, anxiety, depression, sleep disturbances, and muscle aches. These symptoms interact, forming a vicious cycle: dyspnea leads to activity limitation, reduced activity causes a decline in physical capacity, which in turn exacerbates dyspnea. Simultaneously, the decline in physical function triggers psychological distress, and psychological symptoms further reduce activity tolerance and treatment adherence. Symptom clusters in COPD exhibit significant heterogeneity, with different patient populations potentially presenting different symptom combinations and severity levels.

Accurately assessing the impact of COPD is crucial. Disease-specific health status is a core concept, referring specifically to the disease-related symptoms (such as cough, sputum, chest tightness) and their concrete effects on the patient’s functional status (e.g., daily activities, energy levels) (14). Tools like the COPD Assessment Test (CAT) are commonly used in clinical practice to quantify this concept. The CAT focuses on evaluating the core health problems caused by the disease itself, which is related to, yet distinct from, the broader psychosocial construct of quality of life that encompasses factors like stigma and disease burden (14).

Symptom clusters influence patients’ health status through multiple mechanisms (15, 16). Firstly, physical symptoms like dyspnea and fatigue directly limit daily activity capacity. Secondly, psychological symptoms such as anxiety and depression affect social participation and treatment motivation. Finally, the overall burden of symptom clusters may reduce patients’ self-management abilities and treatment adherence. Research (17) indicates that the health status of COPD patients is generally poor. However, current studies on symptom clusters and health status in COPD have several limitations: most employ a cross-sectional design, making it difficult to reveal the dynamic interaction between them; research often focuses on single or a few symptoms, lacking investigation into the overall trajectory of symptom clusters; particularly for the older adults COPD population, in-depth studies on the uniqueness of their symptom experience during pulmonary rehabilitation remain insufficient.

It is particularly important to note that pulmonary rehabilitation, as a dynamic intervention process, may alter the manifestation of symptom clusters and their impact patterns on health status. However, there is currently a lack of in-depth exploration into the evolutionary patterns of symptom clusters across different stages of pulmonary rehabilitation. Different rehabilitation phases may present distinct dominant symptom cluster characteristics, necessitating targeted management strategies. Furthermore, how the strength and pattern of the association between symptom clusters and health status change dynamically during the rehabilitation process, and its underlying mechanisms, have not been fully elucidated.

Based on the above background, this study employs a prospective longitudinal design aimed at systematically investigating the dynamic evolution patterns of symptom clusters and their association mechanism with disease-specific health status in older COPD patients undergoing structured pulmonary rehabilitation. The core objectives of this study are: (1) To identify the core symptom cluster structures at different stages of pulmonary rehabilitation (at enrollment, at 4 weeks, and at 12 weeks); (2) To track the dynamic trajectories and stability of these symptom clusters throughout the entire rehabilitation process; (3) To longitudinally examine the strength of the association between symptom cluster severity and disease-specific health status and its changes over time; (4) To explore potential factors influencing the evolution trajectories of symptom clusters. Based on existing literature and theoretical frameworks, this study proposes the following hypotheses:

*H1*: The symptom cluster structure in older COPD patients exhibits dynamic evolution during pulmonary rehabilitation.

*H2*: A significant positive correlation exists between symptom clusters and disease-specific health status, and the strength of this correlation shifts dynamically as rehabilitation progresses.

*H3*: Adherence to the pulmonary rehabilitation intervention is a significant predictor of improvement in symptom clusters and health status, demonstrating a significant dose-response relationship.

This study expects that by clarifying the dynamic patterns of symptom clusters, it will provide a basis for optimizing staged and precise symptom management strategies. This, in turn, aims to alleviate the symptom burden in older COPD patients, enhance the benefits of pulmonary rehabilitation, and ultimately achieve the goal of improving their health status and long-term prognosis.

## 2 Subjects and methods

### 2.1 Study participants

This study employed a longitudinal design. Patients with COPD were recruited from the respiratory medicine department of Jiangyin People’s Hospital between January 2025 and September 2025. The inclusion criteria were: (1) aged 60–80 years; (2) meeting the diagnostic criteria of the Global Initiative for Chronic Obstructive Lung Disease (GOLD 2023); (3) having received regular maintenance pharmacotherapy for COPD for at least 3 months; (4) normal cognitive function and ability to participate fully in the pulmonary rehabilitation program. The exclusion criteria encompassed: (1) comorbid severe cardiopulmonary diseases such as cardiac function Class III or above, or uncontrolled arrhythmias; (2) having undergone thoracic surgery or hospitalization for acute exacerbation within the past 3 months; (3) presence of contraindications to exercise or psychiatric disorders affecting assessment compliance; (4) a Mini-Mental State Examination (MMSE) score of <24. This study was approved by the Ethics Committee of Jiangyin City, with the approval number 2025-KY020-01. All participants signed written informed consent forms.

### 2.2 Pulmonary rehabilitation intervention protocol

This study implemented a 12-week comprehensive pulmonary rehabilitation program, with home-based rehabilitation as the core component. The program encompassed six dimensions: pharmacological therapy management, functional training, nutritional support, psychological counseling, and home monitoring. The specific details are as follows:

1. Pharmacological Therapy Management: All patients continued their pre-existing regular medication regimen, including bronchodilators (e.g., Tiotropium). A respiratory physician conducted monthly remote follow-ups to assess drug efficacy and adverse reactions, and adjusted the use of inhaled corticosteroids strictly based on indications.
2. Breathing Function Training:Training was conducted twice daily (once in the morning after waking and once before bed). Each session included pursed-lip breathing (5 min) and diaphragmatic breathing (5 min). If patients experienced an acute exacerbation during rehabilitation, postural drainage (assuming a 30° head-down position assisted by family members for percussion) was added.
3. Staged Exercise Training: The exercise protocol followed a principle of gradual progression. Aerobic exercise primarily consisted of indoor walking, starting from 10 min per session and gradually increasing to 30 min, performed twice daily. Strength training involved using 500 mL water bottles for shoulder flexion (10 repetitions/set, 3 sets) and seated leg raises (15 repetitions/set, 2 sets), performed every other day.
4. Nutritional and Hydration Support: The nutritional support implemented in this study was a targeted and enhanced intervention based on an assessment of patients’ habitual baseline dietary patterns. Upon enrollment, most patients exhibited issues such as insufficient protein intake, irregular meal patterns, and *ad hoc* hydration habits. The intervention protocol included: implementing a daily schedule of 5–6 meals (3 main meals supplemented by 2–3 snacks), with a key focus on ensuring increased intake of high-quality protein with each meal (e.g., guaranteeing at least one egg, 100 g of tofu, or fish daily). Patients were explicitly instructed to consume deep-sea fish at least three times per week to address significant baseline dietary gaps. Regarding hydration management, targeting the common habit of drinking only when thirsty, a proactive, scheduled hydration plan was instituted. This involved advising patients to drink 50 mL of water per hour during waking periods and to record daily urine output to quantify adherence. The core objective of this intervention was to correct baseline malnutrition and dehydration risk through structured, supervised nutrition and hydration management, thereby providing the necessary energy substrate for exercise rehabilitation and potentially alleviating fatigue symptoms associated with nutritional status.
5. Psychological and Social Support: With assistance from family members, patients were required to keep a daily mood diary. A study physician provided weekly telephone counseling and psychological support. Additionally, patients were instructed to perform progressive muscle relaxation training (accompanied by light music) for 20 min, three times per week.
6. Home Monitoring and Emergency Response: Patients monitored their resting blood oxygen saturation (SpO₂) and heart rate daily upon waking using a pulse oximeter. Family members assisted in completing the CAT questionnaire assessment once weekly. A pre-established emergency contact protocol was activated immediately if the CAT score increased by ≥3 points compared to the previous assessment.

To assess intervention adherence and explore potential dose–response effects, this study documented patients’ completion of core intervention components (e.g., aerobic exercise, strength training). Intervention adherence was defined as: (number of sessions actually completed/total number of planned sessions) × 100%. Patients with an adherence rate ≥80% were classified into the high-adherence group, while those with an adherence rate <80% were classified into the low-adherence group, to compare the differences in changes in health status (CAT scores) between the two groups. Additionally, Spearman correlation analysis was employed to examine the association between the overall intervention adherence rate and the change in CAT score (ΔCAT = CAT_T0 - CAT_T2).

### 2.3 Assessment instruments

#### 2.3.1 General information questionnaire

A self-designed general information questionnaire was used, primarily collecting data on patient gender, age, marital status, education level, BMI, economic status, etc.

#### 2.3.2 Memorial symptom assessment scale (MSAS)

The MSAS is a self-rated symptom assessment tool comprising two major dimensions. The Symptom Experience Module integrates 29 symptom items: 13 items are derived from the core MD Anderson Symptom Assessment Inventory items (including dyspnea, persistent cough, etc.), with an additional 16 items reflecting characteristic complaints of COPD patients. The Functional Impact Module assesses the degree to which symptoms limit daily functioning, covering six domains including physical activity, emotional regulation, and occupational/household abilities. It employs a 10-point Likert scale (0 = no impact, 10 = extreme distress), quantifying each symptom across three dimensions: frequency, severity, and distress. The total score of the Symptom Experience Module is calculated as the mean of all symptom item scores, resulting in a theoretical total score range of 0–10. A higher total score indicates a greater overall symptom burden for the patient (18–20). This instrument, validated for local use, demonstrates good reliability in populations with chronic airway diseases and can sensitively capture dynamic changes in symptom clusters during pulmonary rehabilitation.

#### 2.3.3 COPD-specific symptom supplementary assessment tool for pulmonary rehabilitation

This tool was developed based on the 2023 ERS/ATS Joint Statement on Pulmonary Rehabilitation (21) and results from multi-center patient interviews, aiming to address the assessment gap in rehabilitation-specific symptoms not fully covered by the MSAS. The questionnaire includes 6 core items across 2 dimensions: ① Physiological Adaptation Symptoms (delayed onset muscle soreness, respiratory-swallowing incoordination, throat irritation associated with breathing training devices); and ② Behavioral Responses to Rehabilitation (decreased exercise adherence, situation-specific respiratory anxiety, diminished rehabilitation confidence). Scoring System and Cut-off Points: The Visual Analogue Scale (VAS) was used to assess symptom distress. The score for each individual item ranges from 0 to 10(0 = no impact, 10 = intolerable). The total score for each dimension (comprising 3 items) ranges from 0 to 30; the total score for the entire instrument (6 items) ranges from 0 to 60. A higher score indicates more severe rehabilitation-related symptom distress. A clear clinical intervention cut-off point was established for this tool: An individualized rehabilitation plan adjustment is triggered when the total score of any dimension reaches ≥15 points, or when the total score of the full scale increases by ≥5 points for two consecutive weeks. This cut-off value was determined based on clinical expert consensus and pre-trial results, aiming to promptly identify a significant symptom burden requiring intervention. In the pre-trial validation (*n* = 62), the scale demonstrated a Content Validity Index (CVI) of 0.85, a significant correlation with rehabilitation dropout rates (*r* = 0.68, *p* < 0.01), and an internal consistency Cronbach’s *α* of 0.77.

#### 2.3.4 COPD assessment test (CAT)

The CAT comprises eight items: cough, phlegm (sputum production), chest tightness, breathlessness upon physical exertion, limitations in daily activities at home, confidence in leaving home, sleep quality, and energy level. Each item is scored using a 5-point Likert scale (0 = no impairment, 5 = severe impairment). The total score ranges from 0 to 40, with a higher score indicating a greater impact of COPD on the patient’s disease-specific health status (22, 23). The CAT demonstrates good construct and criterion validity, and has been widely used and validated in COPD populations. In this study, the scale demonstrated a Cronbach’s *α* coefficient of 0.890, which is consistent with its established good reliability and validity from previous validations in Chinese COPD populations.

### 2.4 Data collection methods

This study employed a prospective longitudinal design. Symptom assessments were conducted at three time points: upon enrollment (T0), at the 4th week of pulmonary rehabilitation (T1), and at the 12th week (T2). Data were collected using validated assessment tools, including the MSAS, the CAT questionnaire, and the COPD-specific symptom supplementary questionnaire. All assessments were performed by uniformly trained respiratory therapists in a dedicated, quiet assessment room within the rehabilitation center to ensure environmental consistency and standardization of data collection. The T0 assessment was conducted within 24 h after the patient provided informed consent. It included the collection of demographic information, clinical baseline data (such as pulmonary function test results), and the initial symptom assessment, taking approximately 25 min to complete. The T1 and T2 assessments were scheduled on Wednesday mornings, 30 min after the group rehabilitation session, using the same set of tools for follow-up evaluation, each requiring about 15 min. For patients unable to attend in-person due to mobility issues, assessments were completed via “video-guided assessment” to maximize the follow-up rate. All collected questionnaire data were independently double-entered by two individuals working back-to-back. Logical checks were performed, and the final database was established using EpiData 3.1 software.

### 2.5 Statistical methods

Data analysis was performed using SPSS 26.0 and Mplus 8.3 software. The distribution of quantitative data was assessed using the Shapiro–Wilk test; data conforming to a normal distribution are described as mean ± standard deviation (x̄ ± s), while qualitative data are described using frequencies and percentages (%).

Symptom Screening Criterion: To focus on clinically prevalent core symptoms, a symptom inclusion criterion was established prior to analysis: only symptoms with an incidence rate ≥ 40% at any of the three time points (T0, T1, or T2) were included in the subsequent symptom cluster analysis. This criterion aimed to ensure that the analyzed symptoms were sufficiently common and representative of the experience of the majority of patients.

Identification and Validation of Symptom Clusters employed the following steps: To identify the symptom cluster structure in older COPD patients during pulmonary rehabilitation (T0, T1, T2), this study used exploratory factor analysis. Factor Analysis Applicability Test: Initially, the suitability of the symptom data (meeting the ≥40% incidence rate criterion at each time point) for factor analysis was evaluated using the Kaiser-Meyer-Olkin (KMO) measure and Bartlett’s test of sphericity. A KMO value > 0.6 and a Bartlett’s test *p*-value < 0.05 were considered indicative of suitability for factor analysis. Factor Extraction and Rotation: Principal component analysis was used to extract initial factors, followed by varimax rotation for orthogonal rotation to achieve a clearer factor structure. Criteria for Factor Retention and Symptom Assignment: The retention of factors was based on the following three criteria: ① Eigenvalue greater than 1; ② Examination of the scree plot inflection point; ③ The rotated factors needed to be clinically interpretable, with each factor containing at least two or more symptom items. The criterion for symptom assignment to a factor was an absolute factor loading ≥ 0.4. If a symptom had loadings ≥ 0.4 on multiple factors (cross-loading), its assignment was determined based on the difference in loadings (< 0.2) and clinical significance, or it was removed. Reliability Assessment: The internal consistency of each identified symptom cluster was assessed using Cronbach’s alpha coefficient, with an *α* > 0.6 considered indicative of acceptable reliability.

Symptom Cluster Trajectory and Correlation Analysis: Dynamic Trajectory Analysis: The consistency of symptom cluster composition across different time points was assessed by calculating the symptom cluster consistency index (number of overlapping symptoms / total number of symptoms × 100%). The stability of the factor structure was evaluated using Procrustes rotation analysis, with a Root Mean Square Error of Approximation (RMSEA) < 0.08 indicating good model fit and structural stability. Correlation Analysis: Spearman’s rank correlation analysis was used to examine the correlations between the severity of symptom clusters (represented by factor scores) and the CAT total score at each time point. To control for inflation of the Type I error rate due to multiple comparisons, a Bonferroni correction was applied to the *p*-values, with a corrected *p* < 0.05 considered statistically significant.

The data analysis in this study primarily adopted a per-protocol analysis approach. Since the effects of pulmonary rehabilitation (e.g., on exercise capacity, symptoms) can diminish significantly after interruption, using an intention-to-treat analysis might underestimate the actual effect of the intervention on participants who completed the program. Therefore, the primary analysis aimed to reveal the dynamic patterns of symptoms and health status in patients who completed the designated rehabilitation plan.

## 3 Results

### 3.1 Patient characteristics

A total of 184 COPD patients aged 60–80 years participating in pulmonary rehabilitation were enrolled and completed the baseline assessment. At the 4-week and 12-week marks of rehabilitation, 178 and 168 valid questionnaires were collected, respectively. During the study period, 16 patients discontinued participation due to hospitalization for acute exacerbations (*n* = 8), loss to follow-up (*n* = 5), or voluntary withdrawal (*n* = 3). A comparison of baseline characteristics between the 16 participants who withdrew mid-study and those who completed the study showed no statistically significant differences in key indicators such as age, gender, and total CAT score (all *p* > 0.05). This suggests that participant withdrawal was random and that the baseline characteristics of the study completers were well-representative of the overall sample. Ultimately, 168 patients completed the entire study assessments. The mean age was 70.3 ± 5.8 years. Detailed characteristics are presented in Table 1.

**Table 1:** Characteristics of COPD patients undergoing pulmonary rehabilitation (*n* = 168).

Characteristic	*n*	Percentage (%)
Gender		
Male	91	54.2
Female	77	45.8
Age group		
60–69 years	76	45.2
70–80 years	92	54.8
Marital status		
Married/Cohabiting	139	82.7
Single/Divorced/Widowed	29	17.3
Education level		
Junior high school or below	67	39.9
High school/Technical secondary school	58	34.5
College degree or above	43	25.6
BMI group (kg/m^2^)		
<18.5 (Underweight)	26	15.5
18.5–24.9 (Normal weight)	106	63.1
≥25 (Overweight/Obese)	36	21.4
Monthly household income per capita (RMB)		
<4,000	93	55.4
4,000–8,000	61	36.3
>8,000	14	8.3

### 3.2 Symptom occurrence and severity at different time points during pulmonary rehabilitation in COPD patients

Based on the pre-defined inclusion criterion (symptom incidence rate ≥ 40%), the analysis revealed a dynamic change in symptom distribution throughout the rehabilitation process. At enrollment (T0), 12 high-frequency symptoms were identified, with incidence rates ranging from 24.5 to 83.7%, dominated by respiratory-metabolic related symptoms. By the 4th week of rehabilitation (T1), the number of high-frequency symptoms increased to 15. It is noteworthy that post-exertional muscle soreness (39.9%) and social situation anxiety (38.2%), despite having incidence rates slightly below the conventional 40% threshold, were included in the T1 analysis due to their clinical relevance and proximity to the threshold. At the 12th week of rehabilitation (T2), there were 13 high-frequency symptoms (incidence rate ≥ 30%), with skin-mucous membrane symptoms and chronic fatigue persisting. The specific incidence rates and severity of each symptom are detailed in Table 2.

**Table 2:** Symptom occurrence and severity at different time points during pulmonary rehabilitation in COPD patients.

Symptom	At enrollment (*n* = 184) (*n* = 184)		At 4 Weeks (*n* = 178)		At 12 Weeks (*n* = 168)	
	Occurrence [*n*(%)]	Severity (x̄ ± s, points)	Occurrence [*n*(%)]	Severity (x̄±s, points)	Occurrence [*n*(%)]	Severity (x̄ ± s, points)
Pain	68(36.9)	3.98 ± 0.76	63(35.4)	3.72 ± 0.69	59(35.1)	3.65 ± 0.62
Fatigue	121(65.8)	5.29 ± 1.29	70(39.3)	4.57 ± 1.02	85(50.6)	4.12 ± 0.95
Nausea	87(47.3)	5.30 ± 1.34	69(38.8)	5.03 ± 1.21	65(38.7)	4.04 ± 0.69
Sleep Disturbance	97(52.7)	5.08 ± 1.09	63(35.4)	4.22 ± 0.88	49(29.2)	3.75 ± 0.82
Distress/Anxiety	123(66.8)	5.35 ± 1.38	109(61.2)	4.73 ± 1.05	68(40.5)	4.12 ± 0.92
Shortness of breath	154(83.7)	5.12 ± 1.65	148(83.1)	4.98 ± 1.25	63(37.5)	3.12 ± 0.82
Forgetfulness	60(32.6)	3.81 ± 0.48	45(25.3)	3.55 ± 0.42	32(19.0)	3.20 ± 0.38
Dry mouth	106(57.6)	4.78 ± 1.11	84(47.2)	4.32 ± 0.97	59(35.1)	3.85 ± 0.79
Sadness	55(29.9)	3.98 ± 0.72	43(24.2)	3.65 ± 0.68	37(22.0)	3.42 ± 0.61
Vomiting	45(24.5)	2.99 ± 0.18	32(18.0)	2.75 ± 0.15	28(16.7)	2.60 ± 0.12
Numbness/Tingling	102(55.4)	4.17 ± 1.07	87(48.9)	3.92 ± 0.98	63(37.5)	3.55 ± 0.85
Cough	159(86.4)	4.89 ± 1.02	136(76.4)	4.57 ± 0.87	63(37.5)	3.12 ± 0.82
Sputum production	148(80.4)	4.92 ± 0.99	121(68.0)	4.35 ± 0.85	84(50.0)	3.45 ± 0.73
Constipation	91(49.5)	4.88 ± 1.12	84(47.2)	4.75 ± 0.98	63(37.5)	3.95 ± 0.85
Diarrhea	89(48.4)	5.42 ± 1.46	86(48.3)	5.08 ± 1.03	79(47.0)	4.09 ± 1.21
Pruritus (Itchy Skin)	106(57.6)	4.89 ± 0.98	113(63.5)	4.57 ± 0.87	92(54.8)	4.18 ± 1.25
Dry skin	93(50.5)	4.82 ± 0.93	94(52.8)	4.32 ± 0.79	90(53.6)	4.09 ± 1.07
Hair loss	77(41.8)	3.19 ± 0.84	73(43.0)	2.98 ± 0.55	66(39.3)	2.87 ± 0.62
Difficulty swallowing	64(34.8)	2.89 ± 0.54	61(34.3)	2.76 ± 0.78	56(33.3)	2.98 ± 0.51
Abdominal bloating	92(50.0)	5.52 ± 1.28	70(39.3)	4.87 ± 0.99	60(35.7)	3.09 ± 0.32
Dizziness	75(40.8)	4.01 ± 1.20	66(37.1)	3.98 ± 1.02	59(35.1)	2.78 ± 0.82
Tinnitus	92(50.0)	3.99 ± 0.91	67(37.6)	2.67 ± 0.88	63(37.5)	2.55 ± 0.75
Blurred vision	89(48.4)	4.11 ± 0.87	71(39.9)	3.70 ± 0.95	69(39.1)	3.18 ± 0.76
Muscle aching	71(38.6)	3.01 ± 0.52	63(35.4)	2.98 ± 0.23	59(35.1)	3.05 ± 0.65
Edema (Limb swelling)	62(33.7)	3.65 ± 0.62	85(47.7)	4.38 ± 1.13	52(30.9)	3.21 ± 0.89
Night sweats	49(26.6)	2.98 ± 0.43	45(25.3)	2.76 ± 0.87	47(28.1)	2.55 ± 0.42
Post-rehab muscle soreness	——	——	71(39.9)	5.97 ± 1.65	81(48.2)	4.02 ± 0.91
Respiratory-swallowing Incoordination	63(34.2)	3.54 ± 0.92	60(33.7)	3.21 ± 0.82	57(33.9)	3.06 ± 0.91
Training aversion	55(29.9)	3.13 ± 0.39	83(46.6)	4.17 ± 0.87	59(35.1)	4.12 ± 0.92
Social situation dyspnea anxiety	61(33.2)	2.98 ± 0.76	68(38.2)	4.38 ± 0.88	62(36.9)	3.98 ± 0.85
Reduced rehabilitation confidence	109(59.2)	5.22 ± 1.26	70(39.3)	3.98 ± 1.01	91(54.2)	4.88 ± 0.76

Post-rehab muscle soreness refers to soreness persisting for 24–48 h after training; Respiratory-swallowing incoordination refers to worsened shortness of breath during eating.

### 3.3 Longitudinal changes and correlational analysis of symptom clusters and **disease-specific health status** during pulmonary rehabilitation in COPD patients

#### 3.3.1 Trends in **disease-specific health status** changes during pulmonary rehabilitation in COPD patients

**Figure 1:**
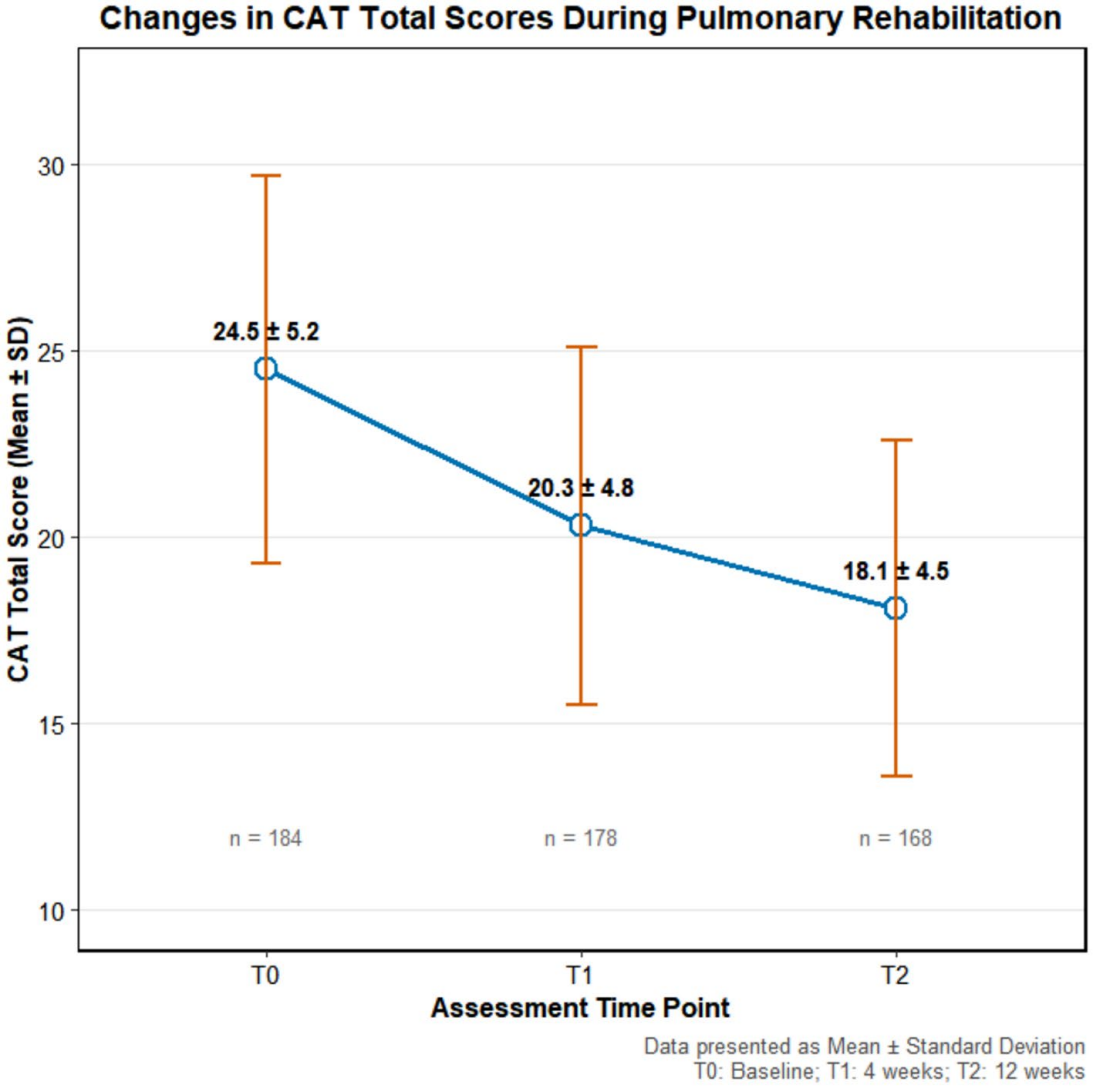
Longitudinal changes in CAT total scores during pulmonary rehabilitation.

Results from the CAT scores indicated that the total score showed a significant improving trend as the rehabilitation program progressed (*F* = 32.72, *p* < 0.001), as shown in Figure 1. Analysis of individual domains revealed that the most pronounced improvements were observed in the Exercise Capacity (T0: 4.5 ± 1.1 points; T2: 3.2 ± 0.9 points) and Energy Level (T0: 4.2 ± 1.0 points; T2: 2.9 ± 0.8 points) domains. However, the Emotional Function (T0: 3.8 ± 1.2 points; T2: 3.5 ± 1.1 points) and Sleep (T0: 3.6 ± 1.0 points; T2: 3.4 ± 0.9 points) domains still maintained relatively high scores even at the T2 time point, suggesting that psychological and sleep-related issues remain key challenges affecting disease-specific health status during the later stages of rehabilitation. See Table 3.

**Table 3:** Changes in CAT scores during pulmonary rehabilitation (Mean ± SD, points).

CAT item	T0 (*n* = 184)	T1 (*n* = 178)	T2 (*n* = 168)	*F* value	*p*-value
Total score	24.5 ± 5.2	20.3 ± 4.8	18.1 ± 4.5	35.72	<0.001
Cough	3.2 ± 1.0	2.8 ± 0.9	2.5 ± 0.8	12.45	<0.001
Sputum (Phlegm)	3.0 ± 0.9	2.7 ± 0.8	2.4 ± 0.7	10.89	<0.001
Chest tightness	3.4 ± 1.1	2.9 ± 1.0	2.7 ± 0.9	15.32	<0.001
Exercise capacity	4.5 ± 1.1	3.5 ± 1.0	3.2 ± 0.9	28.76	<0.001
Daily activities	4.0 ± 1.2	3.3 ± 1.1	3.0 ± 1.0	22.13	<0.001
Emotional function	3.8 ± 1.2	3.6 ± 1.1	3.5 ± 1.1	3.21	0.042
Sleep	3.6 ± 1.0	3.5 ± 1.0	3.4 ± 0.9	1.95	0.146
Energy level	4.2 ± 1.0	3.3 ± 0.9	2.9 ± 0.8	25.67	< 0.001

Differences in CAT scores across time points were compared using repeated-measures analysis of variance (ANOVA). T0: Baseline assessment; T1: 4-week assessment; T2: 12-week assessment.

#### 3.3.2 Characteristics of symptom clusters and their correlation with disease-specific health status at different time points during COPD pulmonary rehabilitation

Exploratory factor analysis combined with varimax rotation was performed on symptoms with an occurrence rate of ≥40% during pulmonary rehabilitation to extract the symptom cluster structure. The Kaiser-Meyer-Olkin (KMO) measure of sampling adequacy yielded values of 0.815 at T0, 0.762 at T1, and 0.703 at T2, and Bartlett’s test of sphericity was significant (*p* < 0.001) at all time points, indicating the suitability of the data for factor analysis. Ultimately, five, five, and four symptom clusters were extracted at T0, T1, and T2, respectively, with cumulative variance contribution rates of 61.32, 59.87, and 57.43%, indicating that the extracted common factors adequately represented the original symptom information.

**Figure 2:**
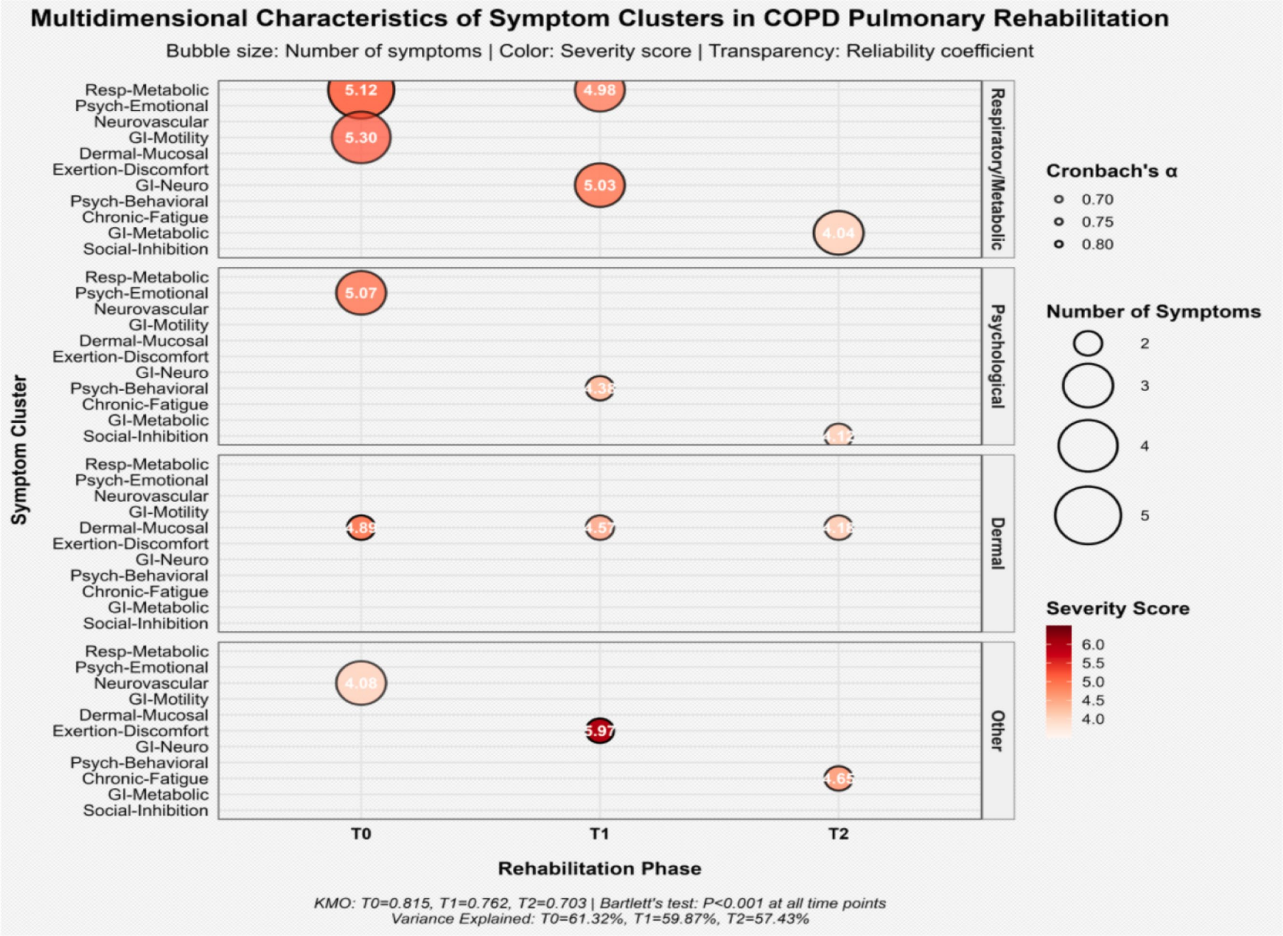
Bubble plot illustrating the multidimensional characteristics of symptom clusters in COPD patients during pulmonary rehabilitation. Bubble size represents the number of symptoms within each cluster (larger bubbles indicate a greater number of symptoms). Bubble color represents the severity score of the symptom cluster (darker/redder colors indicate higher severity).

Internal consistency reliability analysis of the symptom clusters showed that Cronbach’s *α* coefficients ranged from 0.664 to 0.822, indicating stable internal structure and good measurement reliability for the clusters (see Table 4). Longitudinal observation revealed that the composition and severity of the symptom clusters evolved dynamically throughout the rehabilitation process. At T0, patients primarily exhibited a multidimensional symptom burden centered on the Respiratory-Metabolic symptom cluster (Severity: 5.12 ± 1.65 points) and the Psychological-Emotional symptom cluster (5.07 ± 1.09 points). By T1, the severity of the Post-Exertional Malaise symptom cluster was most prominent (5.97 ± 1.65 points), while some of the original psychological-emotional symptoms transformed into a Psychobehavioral symptom cluster. By the final rehabilitation stage (T2), the number of clusters consolidated into four, with the Social Function Inhibition cluster (4.12 ± 0.92 points) and the Chronic Fatigue cluster (4.65 ± 0.82 points) becoming the primary manifestations, reflecting the core challenges in the late stage of rehabilitation. See Table 4 and Figure 2.

**Table 4:** Characteristics of symptom clusters and their correlation with disease-specific health status at different time points during COPD pulmonary rehabilitation.

Time point	Symptom cluster	Cronbach’s α	Severity (x̄ ± s, points)	Key symptoms	Correlation with CAT total score (*r* value/*p*-value)	Strongest correlation with CAT domains
T0 (Baseline)	Respiratory-metabolic cluster	0.763	5.12 ± 1.65	Shortness of breath, cough, sputum, dry mouth, fatigue	0.682/*p* < 0.001	Cough (*r* = 0.712), Shortness of breath (*r* = 0.698)
	Psychological-emotional cluster	0.692	5.07 ± 1.09	Anxiety, reduced rehab confidence, sleep disturbance	0.714/*P* < 0.001	Emotional (*r* = 0.745), Sleep (*r* = 0.683)
	Neurovascular cluster	0.675	4.08 ± 1.02	Numbness/Tingling, tinnitus, blurred vision	0.445/*p* = 0.002	Energy (*r* = 0.512)
	Gastrointestinal motility cluster	0.664	5.30 ± 1.34	Abdominal bloating, constipation, diarrhea, nausea	0.445/*p* = 0.002	Daily activities (*r* = 0.487)
	Skin-mucous membrane reaction cluster	0.802	4.89 ± 0.98	Pruritus (Itchy skin), dry skin	0.218/*p* = 0.085	Sleep (*r* = 0.325)
T1 (4 Weeks)	Respiratory-metabolic cluster	0.682	4.98 ± 1.25	Shortness of breath, cough, sputum	0.645/p < 0.001	Exercise capacity (*r* = 0.678)
	Post-exertional malaise cluster	0.822	5.97 ± 1.65	Post-rehab muscle soreness, edema	0.723/*p* < 0.001	Exercise capacity (*r* = 0.735), energy (*r* = 0.698)
	Gastrointestinal-neural cluster	0.706	5.03 ± 1.21	Nausea, constipation, diarrhea	0.387/*p* = 0.015	Daily activities (*r* = 0.432)
	Psychobehavioral cluster	0.734	4.38 ± 0.88	Training aversion, social situation dyspnea anxiety	0.598/*p* < 0.001	Emotional (*r* = 0.654), Chest tightness (*r* = 0.587)
	Skin-mucous membrane reaction cluster	0.698	4.57 ± 0.87	Pruritus (Itchy Skin), Dry skin	0.195/*p* = 0.102	Sleep (*r* = 0.298)
T2 (12 Weeks)	Chronic fatigue cluster	0.737	4.65 ± 0.82	Fatigue, Post-Rehab Muscle Soreness	0.587/P < 0.001	Energy (r = 0.712), Exercise Capacity (r = 0.654)
	Gastrointestinal-metabolic cluster	0.742	4.04 ± 0.69	Constipation, nausea, diarrhea	0.631/*p* < 0.001	Daily activities (*r* = 0.598), Energy (*r* = 0.543)
	Social function inhibition cluster	0.689	4.12 ± 0.92	Social situation dyspnea anxiety, reduced rehab confidence	0.691/*p* < 0.001	Emotional (*r* = 0.724), Daily activities (*r* = 0.678)
	Skin-mucous membrane reaction cluster	0.703	4.18 ± 1.25	Pruritus (Itchy Skin), dry skin	0.176/*p* = 0.120	Sleep (*r* = 0.285)

Correlation analysis between symptom clusters and disease-specific health status (CAT scores) revealed key clinical pathways of their impact (Table 4). At T0, the Psychological-Emotional symptom cluster had the strongest correlation with the CAT total score (*r* = 0.714, *p* < 0.001), particularly with the Emotional (*r* = 0.745) and Sleep (*r* = 0.683) domains of the CAT. At T1, the Post-Exertional Malaise cluster showed the highest correlation with the CAT score (*r* = 0.723, *p* < 0.001), primarily affecting patients’ exercise capacity and energy level. By T2, the correlation between the Social Function Inhibition cluster and Disease-specific health status was most significant (*r* = 0.691, *p* < 0.001). Its strong associations with the Emotional (*r* = 0.724) and Daily Activities (*r* = 0.678) domains highlighted the determinant role of psychosocial factors in long-term rehabilitation outcomes. Throughout the rehabilitation period, the Skin-Mucous Membrane Reaction cluster, although persistent, did not show a statistically significant correlation with the CAT total score (*p* > 0.05), suggesting its relatively limited impact on patients’ overall disease-specific health status. See Table 4.

#### 3.3.3 Quantitative analysis of symptom cluster characteristics and patient attribution

To quantitatively assess the prevalence of each symptom cluster within the patient population and their relative dominance in terms of severity, this study analyzed the patient attribution percentage for each cluster and compared the inter-group differences in severity (Table 5).

**Table 5:** Patient attribution and severity comparison of symptom clusters at different time points.

Time point	Symptom cluster	Patient attribution (%) (n/N)	Inter-group severity comparison (*p*-value)	Effect size (Cohen’s *d*)	Clinical interpretation
T0 (Baseline)	**Respiratory-metabolic cluster**	83.7 (154/184)	_	_	Dominant cluster: Highest prevalence, serves as comparison benchmark
	Psychological-emotional cluster	66.8 (123/184)	0.043	0.032	Significant difference but small effect size
	Neurovascular cluster	55.4 (102/184)	< 0.001	0.754	Significant difference, medium effect size
	Gastrointestinal motility cluster	49.5 (91/184)	0.210	0.151	Non-significant difference
	Skin-mucous membrane reaction cluster	57.6 (106/184)	< 0.001	0.161	Significant difference but small effect size
T1 (4 Weeks)	Respiratory-metabolic cluster	83.1 (148/178)	< 0.001	0.692	Significant difference, medium effect size
	**Post-exertional malaise cluster**	39.9 (71/178)	—	—	Prominent cluster: Highest severity, serves as comparison benchmark
	Gastrointestinal-neural cluster	48.3 (86/178)	< 0.001	0.624	Significant difference, medium effect size
	Psychobehavioral cluster	46.6 (83/178)	< 0.001	1.219	Significant difference, large effect size
	Skin-mucous membrane reaction cluster	63.5 (113/178)	< 0.001	0.982	Significant difference, large effect size
T2 (12 Weeks)	**Chronic fatigue cluster**	48.2 (81/168)	_	_	Core cluster: Serves as comparison benchmark
	Gastrointestinal-metabolic cluster	47.0 (79/168)	0.025	0.352	Significant difference, small effect size
	**Social function inhibition cluster**	54.2 (91/168)	0.621	0.061	Core cluster: No significant difference from Chronic Fatigue cluster
	Skin-mucous membrane reaction cluster	54.8 (92/168)	0.718	0.055	Non-significant difference

The “Inter-group Severity Comparison” *p*-value represents the result of the comparison between each symptom cluster and the most severe cluster (in bold) at the corresponding time point. Effect size (Cohen’s *d*) is provided to quantify the magnitude of the difference, where |*d*| ≥ 0.8 indicates a large effect, 0.5 a medium effect, and 0.2 a small effect.

At T0 (Baseline), the Respiratory-Metabolic symptom cluster demonstrated exceptionally high prevalence, affecting 83.7% (154/184) of the patients. Its severity was significantly higher than that of the Neurovascular cluster (*p* < 0.001, *d* = 0.754) and the Skin-Mucous Membrane Reaction cluster (*p* < 0.001), and the difference compared to the Psychological-Emotional cluster was also statistically significant (*p* = 0.043). These findings, encompassing both the breadth of patient distribution and the level of symptom severity, collectively confirm its “dominant” status at baseline.

At T1 (4 weeks of rehabilitation), the landscape of symptom clusters shifted. Although the Post-Exertional Malaise cluster had a relatively lower patient attribution percentage (39.9%), its severity was significantly higher than that of all other symptom clusters at this time point (all *p* < 0.001). Notably, the effect size was particularly large (*d* = 1.219) when compared to the Psychobehavioral cluster, which exhibited the greatest difference. This indicates that although this cluster did not affect the widest range of patients, its symptom intensity was the most prominent, constituting the core challenge during the mid-phase of rehabilitation.

At T2 (12 weeks of rehabilitation), the patient attribution percentages for the Chronic Fatigue cluster and the Social Function Inhibition cluster were comparable and stable (48.2 and 54.2%, respectively). Of note, there was no statistically significant difference in severity between these two clusters (*p* = 0.621), and the effect size was minimal (*d* = 0.061). This suggests that they jointly form a core, stable symptomatic structure with comparable severity levels in the late stage of rehabilitation.

### 3.4 Correlation between intervention adherence and improvement in health status

To assess the relationship between intervention dose and effect, we analyzed the intervention adherence and its correlation with the change in CAT score (ΔCAT) among the 168 patients who completed the entire study. Intervention adherence was calculated as: (Number of exercise training sessions actually completed by the patient/Total number of planned training sessions) × 100%.

The results showed that the average exercise training adherence rate for all patients was (85.3 ± 12.7%). Patients were divided into two groups: a high-adherence group (adherence rate ≥ 80%, *n* = 132) and a low-adherence group (adherence rate < 80%, *n* = 36). Baseline characteristics (e.g., age, gender, lung function) showed no statistically significant differences between the two groups (all *p* > 0.05), indicating comparability.

Longitudinal comparison revealed that by the study endpoint (T2), the CAT scores of both groups showed significant improvement compared to baseline (T0), but the degree of improvement differed significantly between the groups (details in Table 5). The total CAT score in the high-adherence group decreased significantly from (24.8 ± 5.3) points at T0 to (17.5 ± 4.1) points at T2, with a mean improvement (ΔCAT) of (7.3 ± 3.2) points. In contrast, the low-adherence group’s total CAT score decreased from (24.1 ± 5.0) points to (20.1 ± 4.5) points, with a mean improvement of (4.0 ± 2.8) points. An independent samples *t*-test indicated that the improvement in CAT score was significantly greater in the high-adherence group compared to the low-adherence group, and this between-group difference was statistically significant (*t* = 5.82, *p* < 0.001).

To further verify this relationship, Spearman’s rank correlation analysis was performed. The results showed a significant positive correlation between the overall intervention adherence rate and the improvement in CAT score (ΔCAT) (*r* = 0.411, *p* < 0.001). This indicates that higher adherence to the pulmonary rehabilitation program was associated with a greater degree of improvement in disease-specific health status (details in Table 6).

**Table 6:** Comparison of CAT score changes between patients with different intervention adherence (mean ± SD).

Group	*n*	CAT score (Baseline, T0)	CAT score (Week 12, T2)	Change in CAT score (ΔCAT)
High-adherence group	132	24.8 ± 5.3	17.5 ± 4.1	7.3 ± 3.2
Low-adherence group	36	24.1 ± 5.0	20.1 ± 4.5	4.0 ± 2.8

The between-group difference in the change in CAT score (ΔCAT) was compared using an independent samples *t*-test (*t* = 5.82, *p* < 0.001).

### 3.5 Analysis of dynamic trajectories of symptom clusters during COPD pulmonary rehabilitation

To thoroughly investigate the evolutionary patterns of symptom clusters throughout the pulmonary rehabilitation process, this study employed a longitudinal tracking design. The stability of the symptom cluster structure across different time points was assessed by calculating the symptom cluster consistency index and the Root Mean Square Error of Approximation (RMSEA) following Procrustes rotation. Based on their dynamic trajectory characteristics, the symptom clusters were primarily categorized into the following three types (details in Table 7).

**Table 7:** Analysis of dynamic trajectories and stability of symptom clusters during pulmonary rehabilitation in COPD patients.

Symptom cluster	Time comparison	Change in symptom composition (overlapping/total symptoms)	Consistency index (%)	Procrustes RMSEA	Structural stability
Respiratory-metabolic cluster	T0-T1	3/5	60.0	0.052	Stable
Psychological-emotional → behavioral transformation cluster	T0-T1	0/3	0.0	0.142	Unstable
Gastrointestinal motility → metabolic evolution cluster	T0-T2	3/6	50.0	0.073	Stable
Skin-mucous membrane reaction cluster	T0-T1-T2	2/2	100.0	0.028	Stable
Exercise-induced discomfort → chronic fatigue cluster	T1-T2	1/3	33.3	0.115	Unstable
Social function inhibition cluster	T1-T2	2/2	100.0	0.032	Stable

RMSEA: Root Mean Square Error of Approximation. An RMSEA value < 0.08 indicates good model fit, while a value > 0.10 indicates poor fit. The Consistency Index represents the percentage overlap of symptoms comprising a cluster between different time points.

**Figure 3:**
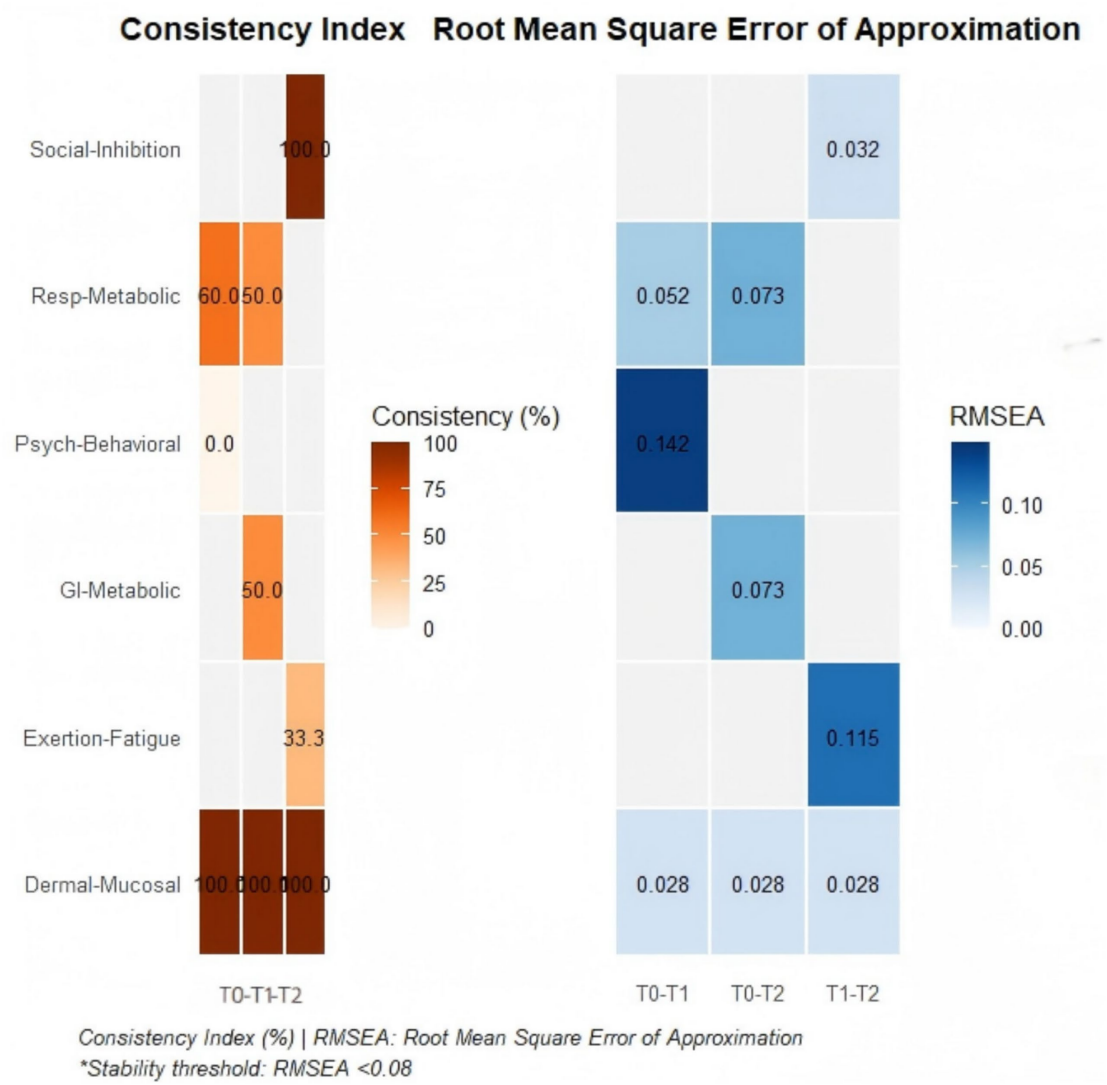
Heatmap of symptom cluster stability and model fit in COPD pulmonary rehabilitation.

High-Stability Symptom Clusters: The composition and structure of these clusters remained highly stable throughout the observation period. The Skin-Mucous Membrane Reaction cluster demonstrated complete consistency (100%) across the entire rehabilitation cycle (T0-T1-T2), with its core symptoms (pruritus, dry skin) remaining unchanged, and exhibited good model fit (RMSEA = 0.028). The Social Function Inhibition cluster, emerging at T1, maintained stable presence of its core symptoms (social situation anxiety, reduced rehabilitation confidence) through to T2 (T1-T2 consistency 100%, RMSEA = 0.032), indicating that psychosocial burden is a persistent key issue during the mid-to-late stages of rehabilitation. See Figure 3.

Structurally Unstable Symptom Clusters: These clusters underwent fundamental restructuring within a short period. During the evolution from T0 to T1, the core symptoms of the Psychological-Emotional cluster completely transformed from “anxiety, reduced confidence” to “training aversion, poor behavioral adherence” (T0-T1 consistency 0%), with significant structural change (RMSEA = 0.142). This suggests that the rehabilitation intervention may have triggered a significant shift in patients’ psychological coping mechanisms.

Compensatory Transformation Symptom Clusters: The core symptoms of these clusters were partially replaced as the condition progressed, but the underlying pathophysiological mechanisms might have continuity. The Gastrointestinal Motility cluster evolved into a Gastrointestinal-Metabolic cluster by T2, shifting its symptomatic presentation from motility disorder features dominated by “abdominal bloating, constipation” to a more prominent manifestation of metabolic disturbance characterized by “nausea, vomiting” (T0-T2 consistency 50.0%). The structural fit for this cluster was acceptable (RMSEA = 0.073), indicating that this evolution might reflect a compensatory adaptation process in response to disease progression or treatment effects.

## 4 Discussion

This prospective longitudinal study dynamically observed the evolutionary trajectories of symptom clusters and their association with Disease-specific health status in 184 older COPD patients undergoing a 12-week pulmonary rehabilitation program. The results reveal that the symptom experience in COPD patients is not isolated but manifests in dynamically evolving clusters. Furthermore, the impact of different symptom clusters on health status varies in focus across different stages of rehabilitation. The following discussion will explore these findings in depth in conjunction with the extant literature.

This study revealed that the respiratory-metabolic symptom cluster (shortness of breath, cough, sputum, dry mouth, fatigue) served as a core cluster present throughout the entire pulmonary rehabilitation period (T0-T2). Its severity showed a slow declining trend with rehabilitation progress but remained at a relatively high level even at T2. This finding is consistent with the study by Chen et al. (24), which indicated that dyspnea and fatigue are the most stable and distressing symptom combination for COPD patients. The underlying reason may be that while pulmonary rehabilitation can improve muscle function and exercise tolerance, it cannot completely reverse the pathological basis of airflow limitation, leading to the persistence of respiratory symptoms. Furthermore, fatigue, as a metabolic byproduct of the high-energy consumption breathing pattern, is tightly coupled with respiratory symptoms (25). This suggests that clinical interventions should not be content with short-term alleviation of respiratory symptoms but need to integrate fatigue management (such as energy conservation techniques and nutritional support) into breathing training for long-term, integrated management.

This study found that the psychological symptom cluster transformed from “emotional distress” (anxiety, reduced confidence) at T0 to “behavioral manifestations” (training aversion, social anxiety) at T1/T2, and interacted with the social function inhibition cluster. Research related to COPD patients has also reported similar psychological transition trajectories (26, 27). The mechanism for this evolution may be that initial anxiety stems from uncertainty about the disease; as rehabilitation progresses, practically encountered difficulties (e.g., breathlessness attacks) transform internal emotions into concrete behavioral avoidance (training aversion) and situational anxiety (social fear). This necessitates that healthcare professionals conduct psychological screening and counseling early in rehabilitation, and introduce cognitive-behavioral therapy in the mid-to-late stages to address specific behavioral barriers, encouraging family involvement to enhance social support (28).

This study observed the symptom cluster shifting from being dominated by acute “post-exertional malaise” (muscle soreness, edema) at T1 to being characterized by “chronic fatigue” as the core at T2. This trajectory reflects the physiological process of the body transitioning from acute stress to chronic adaptation. Exercise-induced discomfort in the early stage of rehabilitation is often a normal reaction to a sudden increase in activity level after long-term disability; if managed inappropriately, it can easily develop into persistent chronic fatigue, becoming a key factor affecting long-term disease-specific health status (29). Therefore, rehabilitation programs must emphasize the principle of gradual progression and educate patients to distinguish between “normal muscle soreness” and “excessive fatigue,” avoiding exercise abandonment due to fear of discomfort while also preventing overtraining.

The gastrointestinal-metabolic symptom cluster evolved from “motility disorder” (bloating, constipation) at T0 to “metabolic disturbance” (nausea, diarrhea) at T2, with a consistency of 50.0%, indicating a possible shift in its pathological mechanism. Early symptoms might be related to low activity levels and medication side effects (e.g., the use of theophylline) (30); later symptoms may be associated with systemic inflammatory response and altered metabolic demands (31). This finding suggests that the assessment of gastrointestinal symptoms in COPD patients should not involve simple symptomatic treatment; instead, the underlying causes need to be explored at different stages of rehabilitation, particularly the assessment and intervention of nutritional and metabolic status (32).

The skin-mucous membrane reaction cluster (pruritus, dryness) and the social function inhibition cluster demonstrated high stability throughout the observation period. The former may be related to the physiological decline of skin function in older adults, chronic hypoxia, and medication effects (33); the latter reveals the profound and persistent impact of COPD as a chronic disabling disease on patients’ social identity and participation capacity. For such stable symptom clusters, routine, preventive management strategies should be established, rather than intervening only after problems arise. The most important finding of this study lies in its first longitudinal demonstration of the dynamic evolutionary patterns of symptom clusters in older COPD patients throughout the entire pulmonary rehabilitation process. Compared to the cross-sectional study by Fei et al. (12), which revealed the static symptom cluster structure at a single time point in stable COPD patients, our data indicate that symptom clusters are not fixed but follow a temporal sequence of ‘respiratory-metabolic dominance → prominent exercise-induced discomfort → psychosocial consolidation’. This discovery breaks through the limitations of traditional cross-sectional research, advancing symptom cluster investigation from static description to a new level of dynamic tracking. Specifically, while the core respiratory-psychological cluster reported by Fei et al. (12) is consistent with our findings at T0, we further observed that with the progression of rehabilitation intervention, this cluster undergoes deconstruction and reorganization: acute exercise-induced discomfort becomes prominent in the mid-phase, while psychosocial factors emerge as the core challenge in the later phase. This evolutionary pattern suggests that pulmonary rehabilitation itself, as a dual physiological and psychological stressor, actively shapes the manifestation of symptom clusters.

This study demonstrates that patients’ Disease-specific health status (CAT total score) followed a complex trajectory during pulmonary rehabilitation, characterized by “initial improvement (T0-T1) - minor fluctuation (T1-T2) - long-term consolidation (T2).” The significant improvement in the T0-T1 phase was primarily driven by rapid gains in respiratory symptoms and exercise capacity, consistent with the short-term benefits reported in international pulmonary rehabilitation guidelines (34). However, the T1-T2 phase entered a plateau period, where improvement in the social/family circumstances dimension was particularly slow and even showed some fluctuation. This phenomenon indicates that physiological improvement has its limitations, while addressing psychosocial issues requires longer timeframes and more integrated interventions. The consolidation of health condition at T2 depended on the effective management of “late-stage core symptom clusters” such as chronic fatigue and social function inhibition. This challenges the traditional pulmonary rehabilitation model that prioritizes physiological aspects over psychosocial components, emphasizing that psychosocial rehabilitation should be a key focus during the mid-to-late stages of intervention.

Longitudinal correlation analysis revealed a dynamic shift in the impact of symptom clusters on health condition, which is a key finding of this study. At T0, the psychological-emotional symptom cluster had the strongest correlation with the CAT total score (*r* = 0.714), highlighting the primary importance of emotional support immediately after diagnosis. By T1, the post-exertional malaise cluster became the predominant factor (*r* = 0.723), making the precision and safety of rehabilitation guidance at this stage crucial, as it directly affects patients’ adherence. By T2, the social function inhibition cluster showed the highest correlation with health condition (*r* = 0.691), this indicates that the ultimate goal of rehabilitation should be the reconstruction of social roles and the restoration of life meaning, rather than merely the improvement of physiological functions.

These findings offer crucial insights for optimizing both clinical practice and public health strategy for the aging population with COPD. At the clinical assessment level, moving beyond a reliance on physiological indicators like FEV1%, we recommend establishing a phased, multidimensional symptom cluster evaluation system. This system would dynamically monitor the evolving symptom profiles of older patients from the point of admission, enabling timely and precise interventions. This refined assessment approach facilitates a fundamental shift in intervention philosophy—from a one-size-fits-all model toward a dynamic strategy of addressing the core symptom clusters for specific patient subgroups at the appropriate time. Based on our longitudinal findings, we propose a staged management framework that integrates clinical care with public health support system. Initial Rehabilitation Phase (T0, “Respiratory-Metabolic Dominance” Stage): For patients presenting with the core respiratory-metabolic cluster, clinical interventions should prioritize optimizing bronchodilator therapy and introducing energy conservation techniques. From a public health perspective, this stage underscores the critical importance of early case-finding and assessment in primary care settings. Strengthening the capacity of community health centers to perform initial screenings and referrals can ensure older adults with COPD enter structured rehabilitation programs before severe deconditioning sets in, thereby improving long-term outcomes and reducing future healthcare utilization. Mid-Rehabilitation Phase (T1, “Post-Exertional Malaise Prominence” Stage): This phase demands proactive management of exercise-induced symptoms to maintain adherence. Clinicians should educate patients on distinguishing normal adaptive responses from concerning signs. Public health initiatives can amplify this by supporting the development of accessible, supervised community-based exercise programs and training for community health workers. This creates a supportive infrastructure that extends the reach of formal rehabilitation, promoting long-term physical activity maintenance—a key goal of healthy aging policies. Late Rehabilitation Phase (T2, “Psychosocial Consolidation” Stage): The emergence of chronic fatigue and social inhibition as core challenges necessitates integrating psychosocial support into care. Interventions should include cognitive-behavioral therapy and peer support groups. This finding has profound public health implications, highlighting the need to move beyond a purely biomedical model. Public health strategies should prioritize integrating mental health services into chronic disease management and fostering community-based senior activity centers. Such initiatives address the social determinants of health in older adults, promoting social participation and functional ability—core components of “healthy aging”—and potentially reducing the burden of disability and isolation on the healthcare system. In summary, the implementation of this dynamic management framework requires a coordinated effort between clinical services and public health systems. By aligning clinical interventions with supportive community-based policies and resources, we can create a sustainable, integrated ecosystem of care that effectively addresses the evolving needs of the growing population of older adults with COPD.

This study has several limitations that need to be fully considered when interpreting the results. Firstly, the study participants were all recruited from a single region, which may limit the representativeness of the sample and introduce potential selection bias. Secondly, symptom assessment primarily relied on patient self-report. Future research could incorporate objective physiological indicators (such as inflammatory marker levels, accelerometer data) for multimodal validation to enhance the reliability of the findings. Furthermore, as an observational study, the temporal management strategy proposed herein requires further validation through randomized controlled trials to confirm its effectiveness.

Another limitation is that the data analysis did not adhere to the ITT principle and excluded patients who withdrew midway, for instance, due to hospitalization for acute exacerbations. Consequently, the conclusions of this study are more applicable to clinically stable patients who can complete the full course of pulmonary rehabilitation intervention. Although this selection helps clearly demonstrate the actual effects of the rehabilitation program on adherent patients, it may introduce selection bias and potentially overestimate the effectiveness of pulmonary rehabilitation in the broader population. Future studies should include a wider range of patient types and employ ITT analysis to provide more generalizable evidence.

Additionally, this study excluded patients with comorbid dementia or severe cognitive impairment. While this exclusion was methodologically necessary to ensure patients could reliably complete the self-report measures, it also limits the generalizability of the findings to older COPD patients with cognitive impairment. The symptom experience, reporting ability, and response to interventions may differ significantly in this population. Future research needs to develop assessment tools suitable for this group (e.g., caregiver-reported scales) or alternative research paradigms to explore their symptom management and rehabilitation needs in depth.

It is also important to note that the symptom improvements observed in this study (e.g., reduced fatigue) resulted from the combined effect of the comprehensive intervention package (including exercise training and nutritional support). Although nutritional interventions, such as increased protein intake and regular hydration, likely provided foundational support for alleviating fatigue by improving energy metabolism and body hydration status, the current results cannot strictly distinguish the individual contributions of exercise and nutrition. Future research employing factorial designs could further clarify the specific contributions of different intervention components to fatigue relief.

Finally, this study used a single-group pre-post test design without a control group (e.g., patients receiving usual care or basic education only), making it impossible to fully rule out the effects of confounding factors such as the natural progression of the disease or the Hawthorne effect. Although the changes in symptom clusters showed a clear temporal association with the rehabilitation phases, the aforementioned factors may still influence the results. Future studies utilizing randomized controlled trial designs will help verify the causal effect of pulmonary rehabilitation on symptom clusters.

## 5 Conclusion

This longitudinal study confirms that symptom clusters in older adults with COPD undergo a predictable dynamic evolution during pulmonary rehabilitation, profoundly influencing their disease-specific health status in stage-specific ways. The trajectory, characterized by a shift from physical to psychosocial dominance, reveals that effective management must be equally dynamic and personalized. Beyond immediate clinical implications, these findings carry substantial significance for public health planning aimed at healthy aging. They provide a compelling rationale for moving from episodic, clinic-centric rehabilitation models toward continuous, community-based support systems that can proactively address the evolving needs of older adults with chronic conditions. By doing so, we can better promote functional ability, sustain rehabilitation gains, and alleviate the escalating societal burden of chronic disease in an aging global population.

## Data Availability

The raw data supporting the conclusions of this article will be made available by the authors, without undue reservation.
