# Correction: A longitudinal study on the correlation between symptom clusters and disease-specific health status in older adults with chronic obstructive pulmonary disease during pulmonary rehabilitation

**DOI:** 10.3389/fpubh.2026.1797965

**Published:** 2026-02-09

**Authors:** Jinghua Yang, Na Xu, Mengyao Liang

**Affiliations:** 1 Department of Nursing, Affiliated Jiangyin Hospital of Nantong University, Jiangyin, China; 2 Department of Respiratory Critical Medicine, Affiliated Jiangyin Hospital of Nantong University, Jiangyin, China; 3 Department of Nursing, The Sixth People's Hospital of Nantong, Nantong, China

**Keywords:** aged, health status, longitudinal studies, pulmonary disease, chronic obstructive, symptom clusters

Author Jinghua Yang was erroneously assigned as corresponding author. The correct corresponding authors are Na Xu and Mengyao Liang.

The original version of this article has been updated.

